# Case Report: Identification of Multiple TERT and FGFR2 Gene Fusions in a Pineal Region Glioblastoma Case

**DOI:** 10.3389/fonc.2021.739309

**Published:** 2021-12-16

**Authors:** Xing Guo, Teng Chen, Shiming Chen, Chao Song, Dezhi Shan, Shujun Xu, Shuo Xu

**Affiliations:** ^1^ Department of Neurosurgery, Qilu Hospital, Cheeloo College of Medicine and Institute of Brain and Brain-Inspired Science, Shandong University, Jinan, China; ^2^ Shandong Key Laboratory of Brain Function Remodeling, Jinan, China; ^3^ Department of Pathology, Qilu Hospital, Shandong University, Jinan, China; ^4^ State Key Laboratory of Translational Medicine and Innovative Drug Development, Jiangsu Simcere Diagnostics Co., Ltd, Nanjing, China

**Keywords:** glioblastoma, somatic variation, TERT, FGFR2, amplification, gene fusion

## Abstract

As an oncogenic somatic variant, telomerase reverse transcriptase promoter (TERTp) mutations are frequently observed in adult glioblastoma (GBM). Alternatively, we report the first case of glioblastoma with TERT amplification accompanied by multiple TERT and FGFR2 gene fusions instead of TERTp mutation. A 55-year-old woman presented with dizziness, headache, and diplopia for three weeks. Magnetic resonance imaging (MRI) demonstrated a heterogeneously enhancing lobulated mass centered in the pineal region. Partial tumor resection and ventriculoperitoneal shunt were achieved, and the residual tumor was then treated with standard radiation. The tumor was diagnosed as GBM, IDH-wild type, WHO grade IV, and the Ki67 proliferation index was high (30–40%). Intriguingly, TERT amplification without TERTp mutation was identified *via* next generation sequencing (NGS). Further analysis revealed multiple TERT (TERT–NUBPL, MARCH6–TERT, and CJD4–TERT) and FGFR2 (CXCL17–FGFR2, SIPA1L3–FGFR2, FGFR2–SIPA1L3, and FGFR2–CEACAM1) gene fusions. After the surgery, the patient’s condition deteriorated rapidly due to the malignant nature of the tumor and she died with an overall survival of 3 months. Our report provides the molecular clue for a novel telomerase activation and maintenance mechanism in GBM.

## 1 Introduction

Somatic variants in human glioblastoma (GBM) are increasingly being recognized as a potent driver of tumorigenesis and progression (1). Apart from necessary determination of isocitrate dehydrogenase (IDH) status and chromosomal 1p/19q co-deletion, several other markers can be considered for routine examination, namely, assessment of telomerase reverse transcriptase promoter (TERTp), B-Raf (BRAF), and H3F3A mutations (2).Among these diagnostic and prognostic indicators, mutated TERTp is frequently observed in adult IDH-wild-type GBM, which involves telomere stabilization, tumor immortality, and progression (3–5). On the other hand, the importance of chromosomal translocations leading to oncogenic fusions has been demonstrated in GBM. For instance, aberrant fibroblast growth factor receptor (FGFR) activation, caused by oncogenic fusions, triggers oxidative phosphorylation and mitochondrial biogenesis, highlighting this pathway as a therapeutic target for GBM (6). Although coexistence of TERTp mutation and FGFR3–TACC3 (transforming acidic coiled-coil-containing protein3) fusion has been described in adult GBM, telomerase reverse transcriptase (TERT) fusion itself has been seldomly reported for glioma yet (7–9).

Herein, we report a case of TERT amplification, accompanied by multiple TERT and FGFR2 fusion in a GBM located in the pineal region, which was validated by next generation sequencing (NGS) analysis. This finding may provide insights into a new telomere maintenance mechanism and a potential therapeutic target for GBM.

## 2 Case Presentation

**Figure 1:**
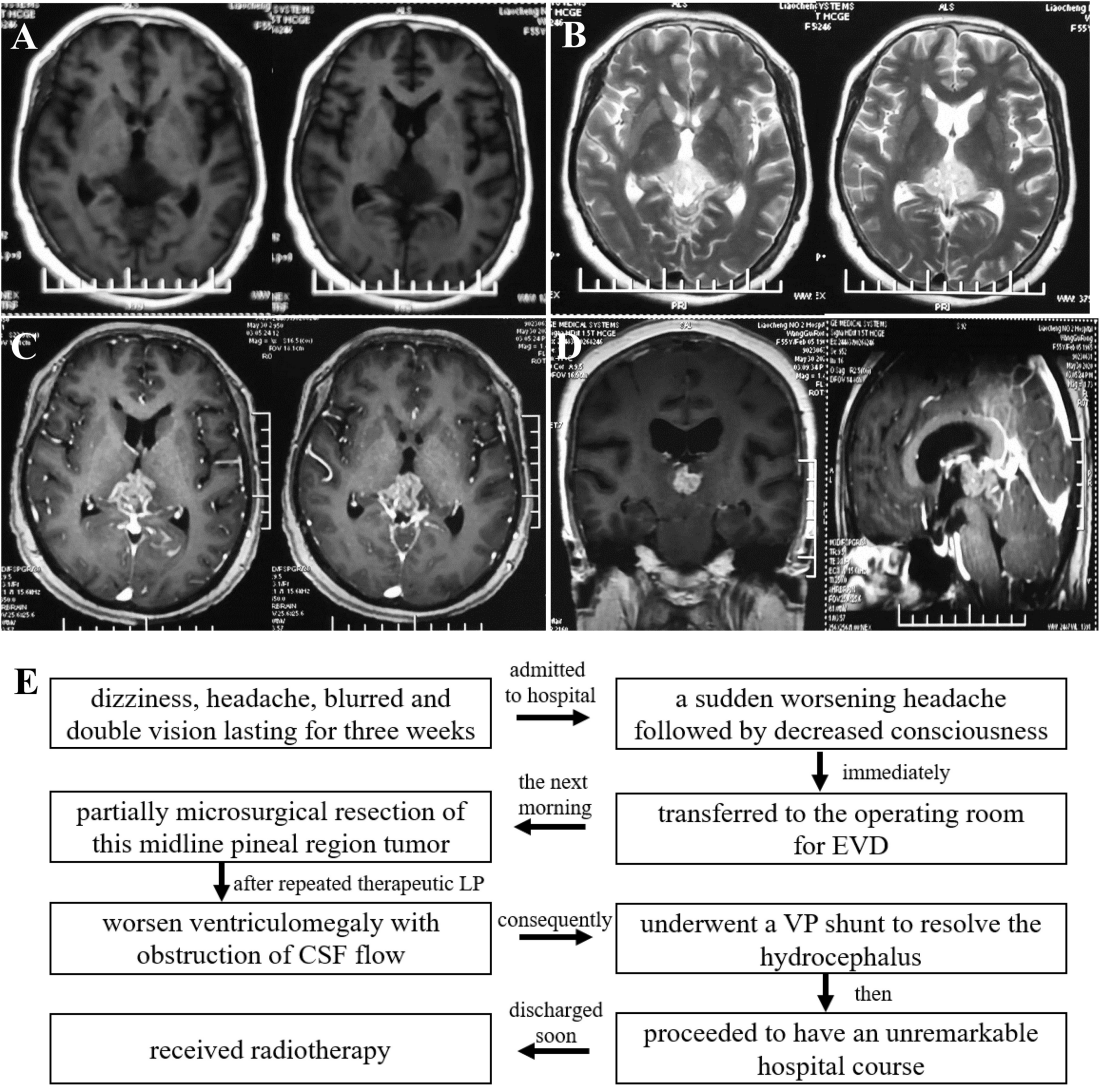
Pre-operative T2-weighted **(A)**, T2-FLAIR weighted **(B)**, and enhanced axial, coronal, sagittal T1-weighted **(C, D)** magnetic resonance images showed a heterogeneously enhancing solid mass in pineal gland region, extending into the bilateral thalamus and obliterating the third ventricle, with ventriculomegaly. A flow chart showcased the timeline with relevant data from the episode of care **(E)**.

A 55-year-old female patient complained of dizziness, headache, blurred and double vision lasting for three weeks. Physical examination confirmed the existence of Parinaud syndrome, including up gaze palsy and convergence-retraction nystagmus. Magnetic resonance imaging (MRI) further confirmed a heterogeneously enhancing lobulated mass extending into the bilateral thalamus and obliterating the third ventricle, with associated ventriculomegaly (**Figures 1A–D**).

After being admitted to the hospital, she developed a sudden worsening headache followed by a decreased consciousness. The patient was immediately transferred to the operating room for external ventricular drainage (EVD) *via* a frontal burr-hole. Lateral supracerebellar infratentorial approach was performed the next morning for microsurgical resection of this midline pineal region tumor. Unfortunately, the tumor was partially removed because of copious bleeding. Her symptoms did not improve significantly after repeated therapeutic lumbar punctures (LP). Cerebrospinal fluid (CSF) flow study demonstrated the worsening ventriculomegaly with obstruction of CSF flow. Consequently, she underwent a ventriculoperitoneal (VP) shunt to resolve the hydrocephalus. Then she proceeded to have an unremarkable hospital course and was discharged soon (**Figure 1E**).

**Figure 2:**
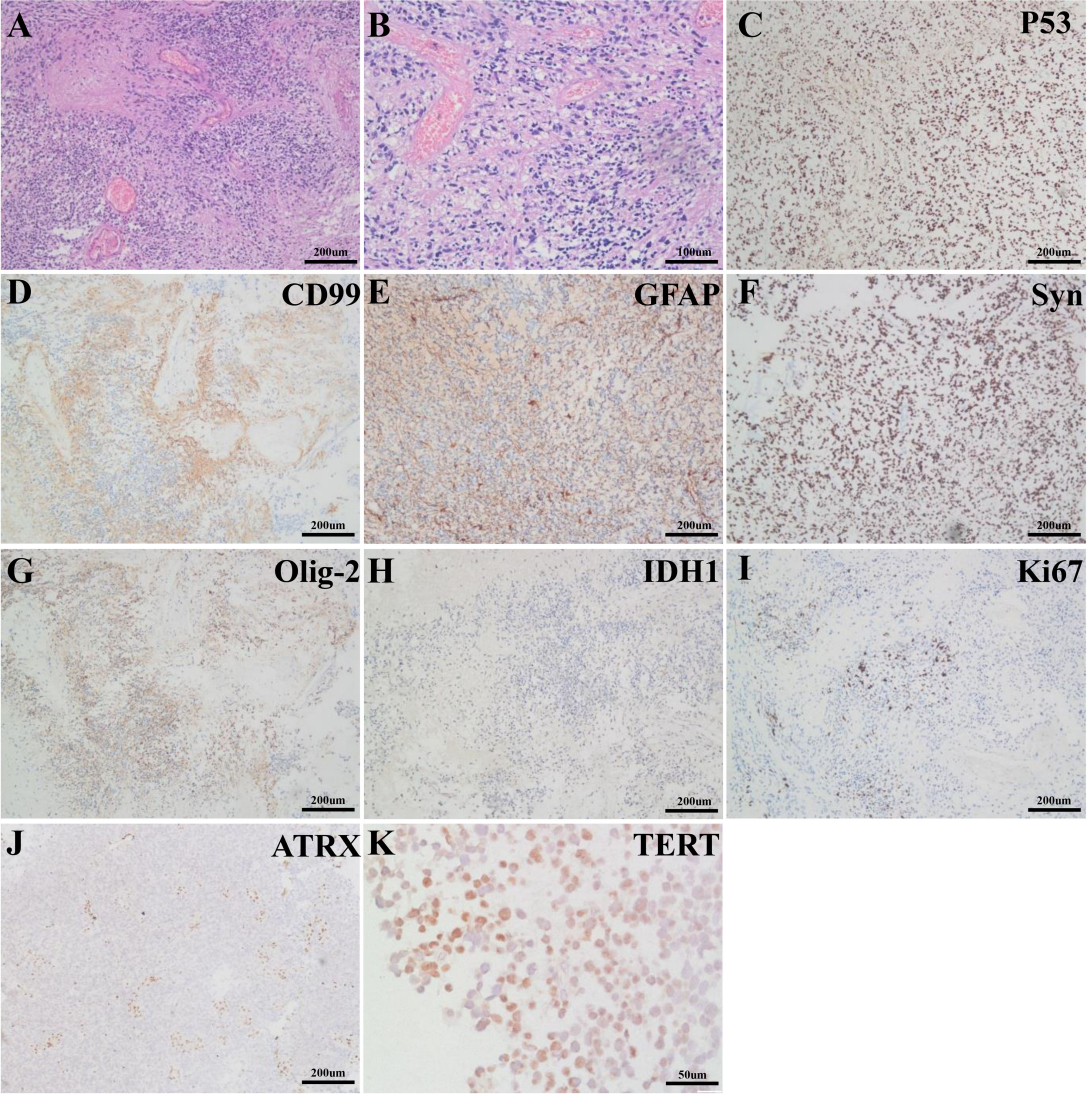
H&E-stained sections showed the tumor was comprised of atypical glial tumor cells with vascular proliferation and necrosis **(A)**. Mitotic figures and perivascular pseudorosettes were easily identified **(B)**. Immunohistochemical sections presented positive expression of P53 (**C**, ZM-0408, ZSGB−BIO), CD99 (**D**, ZM-0296, ZSGB−BIO), GFAP (**E**, MXB Biotechnologies), Syn (**F**, ZA-0506, ZSGB-BIO), Olig-2 (**G**, ZA-0561, ZSGB-BIO), IDH1 (**H**, ZM-0447, ZSGB-BIO) and negative expression of ATRX (**J**, ZA-0016, ZSGB-BIO) in the tumor. The Ki-67 proliferative index was 30–40% (**I**, H10501, Ventata). In addition, TERT (**K**, TA301588, ZSGB-BIO) was substantially expressed in the tumor.

H&E-stained sections showed high density of tumor cells with elevated mitotic activity, vascular proliferation, necrosis with pseudopalisading, and perivascular pseudorosettes (**Figures 2A, B**). Immunohistochemical staining was performed and demonstrated that the tumor cells expressed P53 (**Figure 2C**), CD99 (**Figure 2D**), GFAP (glial fibrillary acidic protein, **Figure 2E**), synaptophysin (Syn, **Figure 2F**), Olig-2 (**Figure 2G**), and IDH1 (**Figure 2H**). The Ki-67 proliferative index was high, approximately 30–40% (**Figure 2I**). In addition, the ATRX was negatively expressed in tumor cells (**Figure 2J**). In conclusion, the histological diagnosis was GBM.

**Figure 3:**
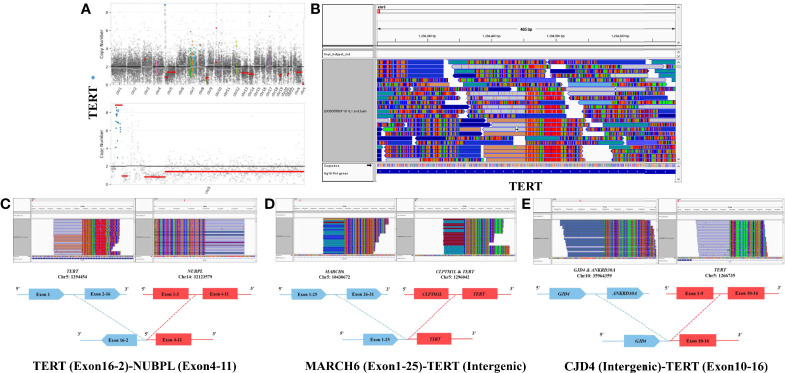
Copy number variation (CNV) of the TERT gene was high in the whole genome **(A)**. TERT gene was amplified 8.94-fold on chromosome 5 **(A)**. Next generation sequencing (NGS) revealed significant TERT gene rearrangement **(B)**, including multiple TERT (Exon16-2)–NUBPL (Exon4-11) **(C)**, MARCH6 (Exon1-25)–TERT (Intergenic) **(D)**, and CJD4 (Intergenic)–TERT (Exon10-16) **(E)** fusions.

**Figure 4:**
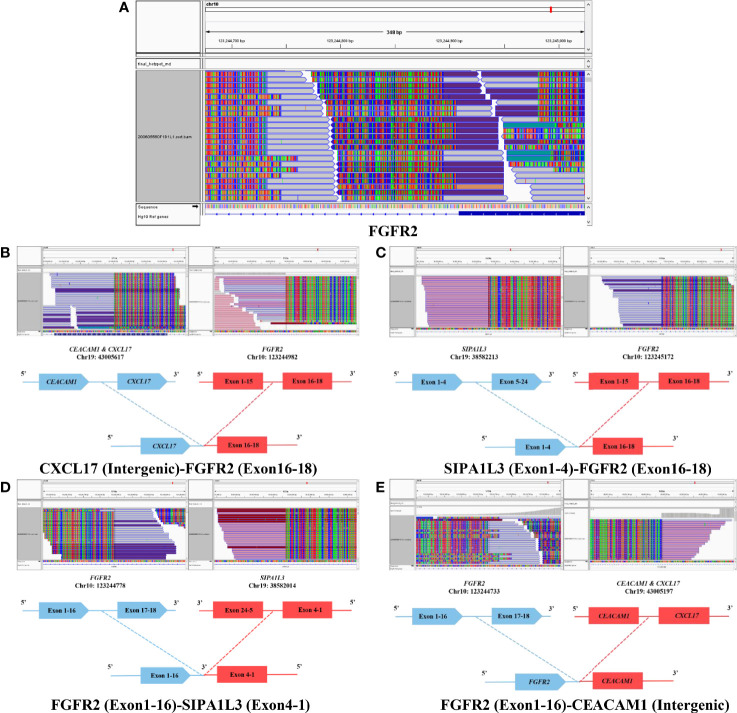
Next generation sequencing (NGS) revealed significant FGFR2 gene rearrangement **(A)**, including multiple CXCL17 (Intergenic)–FGFR2 (Exon16-18) **(B)**, SIPA1L3 (Exon1-4)–FGFR2 (Exon16-18) **(C)**, FGFR2 (Exon1-16)–SIPA1L3 (Exon4-1) **(D)** and FGFR2 (Exon1-16)–CEACAM1 (Intergenic) **(E)** fusions.

To further clarify its molecular characteristics, a comprehensive genomic profiling was performed using a 131 cancer-related genes panel based on NGS analysis *via* DNA-based hybrid capture in formalin-fixed and paraffin-embedded primary tumor tissue (**Additional File 1**). Remarkably, the NGS analysis revealed the presence of TERT amplification (~8.94 fold) without TERTp mutation. Further sequencing showed presence of multiple TERT fusions [TERT (Exon16-2)–NUBPL (Exon4-11), MARCH6 (Exon1-25)–TERT (Intergenic) and CJD4 (Intergenic)–TERT (Exon10-16), **Figure 3**] and also FGFR2 fusions [CXCL17 (Intergenic)–FGFR2 (Exon16-18), SIPA1L3 (Exon1-4)–FGFR2 (Exon16-18), FGFR2 (Exon1-16)–SIPA1L3 (Exon4-1), and FGFR2 (Exon1-16)–CEACAM1 (Intergenic), **Figure 4**], both of which were rarely reported previously. CDKN2A/CDKN2B loss, TP53 mutation (p.H179R), and 19q chromosome deletion were also positive (data not shown). In contrast, IDH1, IDH2, BRAF V600E, H3F3A, HIST1H3B, HIST1H3C, ATRX, EGFR, RELA, YAP1, MYB, and NF1 mutations were not identified. Also, O (6)-methylguanine DNA methyltransferase (MGMT) promoter was unmethylated. The final diagnosis was GBM, IDH-wild type, WHO grade IV, based on the 2016 World Health Organization (WHO) classification of central nervous system (CNS) tumors and cIMPACT-NOW workgroup.

After the discharge, the patient received radiotherapy. Considering the unfavorable molecular profiling and MGMT promoter methylation status, we advised the attempt of Pemigatinib, a potent inhibitor of FGFRs, but she refused and decided to receive temozolomide chemotherapy. Unfortunately, her condition deteriorated rapidly due to the malignant nature of the tumor. The patient eventually died with an overall survival of 3 months.

## 3 Discussion and Conclusions

Glioma accounted for 17% of tumors in the pineal region (10). As pineal region glioma is rare, its biological behavior, molecular pathology, and therapeutic strategy were not well characterized. Complete surgical resection of pineal region tumors correlated with favorable survival; however, the outcome of pineal region high-grade gliomas seemed to be independent of the extent of surgical resection (11). In the revised 2016 WHO Classification of Tumors of the CNS, H3K27M-mutant diffuse midline glioma, arising in the thalamus, pons, and spinal cord, was classified as a WHO grade IV glioma. The genetic landscape studies revealed that pineal region gliomas also displayed certain features of diffuse midline glioma with H3K27M-mutation (12). Although without H3K27M mutation, this pineal region glioma was defined as GBM, WHO grade IV. The aggressive and invasive behavior, impossibility of completing tumor resection, and also molecular profiling (IDH-wild type and TERT amplification), indicated an unfavorable outcome.

Molecular profiling was formally introduced to glioma in the revised 2016 WHO Classification of Tumors of the CNS, namely IDH and H3K27M mutation. Other well-known molecular markers include mutated TERTp, alpha-thalassemia/mental retardation syndrome X-linked (ATRX), BRAFV600E, TP53, and EGFR. Numerous studies show that TERT activity plays a central role in the unlimited self-renewal potential of cancer cells *via* regulating telomerase activity (13). TERTp mutation is ascertained to give rise to the reactivation of TERT in multiple cancers, including glioma (14). Intriguingly, TERT amplification was observed in this case secondary to TERT gene fusions (TERT–NUBPL, MARCH6–TERT and CJD4–TERT) instead of TERTp mutation. Among the three TERT fusions, TERT–NUBPL and CJD4–TERT seem to be not expressed and MARCH6–TERT fusion is the most possible to produce functional protein. To further verify the influence of these TERT fusions, we also examined the TERT protein expression *via* immunohistochemistry. Unsurprisingly, TERT expression is positive in the tumor tissue (**Figure 2K**). Compared to TERTp mutation, TERT gene fusion is quite rare. Limited pieces of literature regarding TERT gene fusion were found in cases with GBM (CCDC127–TERT), clear cell sarcoma (IRX2–TERT fusion), nontranslocation-related sarcomas (TRIO–TERT fusion), spindle cell liposarcoma (CTNND2–TERT fusion), and metastatic Leydig cell tumors (RMST–TERT, LDLR–TERT, and B4GALT5–TERT fusions) (15–19). Moreover, Stransky et al. observed that the samples harboring TRIO–TERT fusions display an elevated TERT mRNA expression level and downstream activities (20). Barthel et al. systematically analyzed the correlation of telomere length and somatic alterations in cancer and provided new insights into the telomerase activity induced by TERT abnormalities including TERT fusions (21). Although the influence of TERT gene fusion in this case is largely unknown, this alternation raises the possibility that this molecular feature might represent a novel regulatory mechanism for telomerase reactivation in GBM.

FGFRs are a highly conserved family of receptor tyrosine kinases (RTK), which are involved in several pathological processes, including cancer. Pathogenic FGFR mutation and fusion are common (~30%) in urothelial carcinoma and cholangiocarcinoma but quite rare in tumors of central nervous system, especially for glioma (22–27) (**Additional File 2**). Notably, FGFR–TACC gene fusions were first discovered in GBM and represent a promising therapeutic target (28). FGFR2 was reported to contribute to glioma proliferation and radiation resistance (29, 30). Until recently, Maria-Magdalena et al. described a case of IDH-mutant GBM harboring FGFR2–TACC2 gene fusion with a 2.5-month survival (26). Coincidentally, the survival of this GBM case with FGFR2 fusions (CXCL17–FGFR2, SIPA1L3–FGFR2, FGFR2–SIPA1L3 and FGFR2–CEACAM1) was also extremely miserable. Among the four FGFR2 fusions, none of them seemed to produce functional fusion proteins, as the functional kinase domains (exons 10–18) in the FGFR2 fusions of this case were not intact. However, this rearrangement of FGFR2 may indicate a DNA genome instability promoting the malignant progression of tumor. Despite the different TERT genetic profiling and FGFR fusion member, the coexistence of TERT mutation and FGFR gene fusion might support a potential pathway to robust tumor progression.

Even with an aggressive multimodal therapy, the average survival of patients with malignant glioma is grim. Therefore, molecular testing is widely encouraged, as patients with a detected driver mutation may respond to a targeted therapy on a compassionate use basis. For instance, a peptide vaccine targeting mutant IDH1 induced potent immune responses in patients with newly diagnosed glioma and exhibited promising therapeutic potential (31). In this case, the patient was diagnosed with FGFR2 gene fusion, who might respond to FGFR2 inhibitors, such as Pemigatinib (32). Although available reports about precision targeting of FGF/FGFR signaling are quite limited for GBM, this approach has shown significant efficacy and safety in the treatment of advanced cholangiocarcinoma and bladder cancer (33). Further studies are necessary to overcome the challenges regarding the penetration ability through the blood–brain barrier and determine the genetic and epigenetic heterogeneity within the glioma microenvironment.

Here, we report the first case of a pineal region GBM with multiple TERT and FGFR2 gene fusions, which sheds light on a novel mechanism of telomerase activation and tumor progression in malignant glioma. In an era of personalized and precision medicine, molecular profiling should play an increasingly important role in the diagnosis and treatment of CNS tumors. Also, further investigation should be conducted to understand the regulatory and therapeutic impacts of TERT and FGFR2 gene fusions for GBM.

## Data Availability Statement

The datasets presented in this study can be found in online repositories. The names of the repository/repositories and accession number(s) can be found in the article/**Supplementary Material**.

## Ethics Statement

The studies involving human participants were reviewed and approved by the Shandong University Qilu Hospital Ethics Committee. The patients/participants provided their written informed consent to participate in this study. Written informed consent was obtained from the individual(s) for the publication of any potentially identifiable images or data included in this article.

## Author Contributions

SX and SJX designed the study. XG collected and analyzed clinical data. SC performed H&E staining and immunohistochemistry of tumor sections. TC and DS gave advices in study design. CS helped with the NGS analysis. All authors contributed to the article and approved the submitted version.

## Funding

This work was supported by grants from the Taishan Scholarship Young Expert Program (tsqn201909174), the National Natural Science Foundation of China (No. 81802966) and Natural Science Foundation of Shandong Province of China (No. ZR2019BH057).

## Conflict of Interest

Author SC was employed by Jiangsu Simcere Diagnostics Co., Ltd.

The remaining authors declare that the research was conducted in the absence of any commercial or financial relationships that could be construed as a potential conflict of interest.

## Publisher’s Note

All claims expressed in this article are solely those of the authors and do not necessarily represent those of their affiliated organizations, or those of the publisher, the editors and the reviewers. Any product that may be evaluated in this article, or claim that may be made by its manufacturer, is not guaranteed or endorsed by the publisher.

## Abbreviations

ATRX, alpha-thalassemia/mental retardation syndrome X-linked; CNS, central nervous system; CSF, Cerebrospinal fluid; EVD, external ventricular drainage; FGFR, fibroblast growth factor receptor; IDH, isocitrate dehydrogenase; MGMT, O (6)-methylguanine DNA methyltransferase; MRI, Magnetic resonance imaging; NGS, next generation sequencing; Syn, synaptophysin; TACC3, transforming acidic coiled-coil-containing protein3; TERT, telomerase reverse transcriptase; TERTp, telomerase reverse transcriptase promoter; VP, ventriculoperitoneal; WHO, World Health Organization.
