# Supplementary material for: Case Report: Identification of Multiple TERT and FGFR2 Gene Fusions in a Pineal Region Glioblastoma Case
Source: Front Oncol. 2021 Dec 16;11:739309. doi: 10.3389/fonc.2021.739309 (PMC8716851; doi:10.3389/fonc.2021.739309)
Supplement: Supplementary file 1 [file Table_1.docx]

| **DNA-based NGS sequencing cancer-related panel of the following 131 genes** | | | | | | | | |
| --- | --- | --- | --- | --- | --- | --- | --- | --- |
| ACVR1 | AKT1 | AKT2 | ALK | APC | AR | ARAF | ARID2 | ATM |
| ATRX | B2M | BCL2L11 | BCOR | BRAF | BRCA1 | BRCA2 | CBL | CCND2 |
| CDK4 | CDK6 | CDKN2A | CDKN2B | CDKN2C | CHEK2 | CIC | CTNNB1 | DAXX |
| DDR2 | DDX3X | DICER | DNMT3A | EGFR | EPCAM | ERBB2 | ERBB3 | ERBB4 |
| ESR1 | FAT1 | FBXW7 | FGF4 | FGFR1 | FGFR2 | FGFR3 | FGFR4 | FLT3 |
| FLT4 | FUBP1 | GNA11 | GNAQ | GNAS | H3F3A | HDAC4 | HIST1H3B | HIST1H3C |
| HMCN1 | HNF1A | HRAS | IDH1 | IDH2 | IRS2 | JAK1 | JAK2 | KDM5A |
| KIT | KLF4 | KRAS | MAP2K1 | MAPK1 | MDM2 | MDM4 | MEN1 | MET |
| MLH1 | MPL | MSH2 | MSH6 | MTOR | MYB | MYC | MYCN | NAB2 |
| NF1 | NF2 | NOTCH1 | NR3C1 | NRAS | NTRK1 | NTRK2 | NTRK3 | PDGFRA |
| PDGFRB | PIK3CA | PIK3CB | PIK3R1 | PLCG1 | PMS2 | POLE | POLR2A | PPM1D |
| PTCH1 | PTEN | PTPN11 | RAF1 | RB1 | RELA | RET | RGPD3 | RICTOR |
| ROS1 | SDHA | SETD2 | SMAD4 | SMARCA4 | SMARCB1 | SMARCE1 | SMO | SRC |
| STAG2 | STAT6 | TERT | TP53 | TRAF7 | TSC1 | TSC2 | USP8 | VEGFA |
| VEGFB | VEGFR1 | KDR | VHL | YAP1 |  |  |  |  |

Additional file 1
