# Supplementary material for: Case Report: Identification of Multiple TERT and FGFR2 Gene Fusions in a Pineal Region Glioblastoma Case
Source: Front Oncol. 2021 Dec 16;11:739309. doi: 10.3389/fonc.2021.739309 (PMC8716851; doi:10.3389/fonc.2021.739309)
Supplement: Supplementary file 2 [file Table_2.docx]

| **No** | **Age** | **Sex** | **Histologic diagnosis** | **FGFR2 gene fusion** | **Location** | **Therapy** | **Follow-up**  **（Month）** | **Status** |
| --- | --- | --- | --- | --- | --- | --- | --- | --- |
| 1(22) | 6 | M | mixed neuronal-glial tumors | FGFR2‑INA | Temporal | NA | NA | NA |
| 2(23) | 9 | F | mixed neuronal-glial tumors | FGFR2‑INA | Parietal | NA | NA | NA |
| 3(24) | 7 | M | ganglioglioma （oligodendroglia） | FGFR2-KIAA1598 | Temporal | GTR | 63 | Alive, progression |
| 4(24) | 35 | M | ganglioglioma （oligodendroglia） | FGFR2-INA | Parietal | NA | 109 | Alive, progression |
| 5(25) | 6 | F | Ganglioglioma | FGFR2-KIAA1598 | Peri-insular | NA | NA | NA |
| 6(26) | 48 | F | Glioblastoma | FGFR2- TACC | Temporal | GTR | 2.5 | Dead |
| 7(27) | 4 | M | low-grade neuroepithelial tumors | GFR2-CTNNA3 | Temporal | GTR | 86 | Alive, NED |
| 8(27) | 9 | M | low-grade neuroepithelial tumors | FGFR2-KIAA1598 | Frontal | GTR | 36 | Alive,  NED |
| 9(27) | 10 | M | low-grade neuroepithelial tumors | FGFR2-KIAA1598 | Occipital | GTR | 62 | Alive,  NED |
| 10 | 55 | M | GBM | FGFR2-CEACAM1, FGFR2-SIPA1L3 | Pineal | PR | 3 | Dead |

Additional file 2: Clinical, histological features and treatment of tumors with FGFR2 gene fusions in central nervous system

NA: not applicable; GTR: gross total resection; PR: partial resection; NED, no evidence of disease.
